# Content, implementation, and evaluation of capacity-strengthening initiatives for public and patient involvement in mental health research: a scoping review protocol informed by implementation science

**DOI:** 10.12688/hrbopenres.14287.2

**Published:** 2026-05-07

**Authors:** Shaakya Anand-Vembar, Brian Keogh, Agnes Higgins, Greg Sheaf, Yulia Kartalova-O’Doherty, Olivia Longe, Lorna Staines, David McEvoy, Allyson J Gallant, Caroline Wilson, Leona Ryan, Christine FitzGerald, Caoimhe Nic Aodha, Chelsea Ryan, Michelle Doody, Nikki Horkan, Nora Hanney, Oisín Breen, Robyn Thomas, Sarah Watters, Louise Doyle, David Cotter, Catherine D Darker, Mary Cannon, John Lyne, Colm McDonald, Colm Healy, Gary Donohue, Sarah Burke, Karen O'Connor, David Mongan, Rebecca Murphy, Donal O'Keeffe

**Affiliations:** 1 Trinity College Dublin School of Nursing and Midwifery, Dublin, Ireland; 2 School of Medicine, University of Galway, Galway, Ireland; 3 University of Dublin, Ussher Library, Trinity College Dublin, Dublin, Ireland; 4 Royal College of Surgeons in Ireland, Dublin, Ireland; 5 Trinity College Dublin Department of Public Health and Primary Care, Dublin, Ireland; 6 School of Psychology, University of Galway, Galway, Ireland; 7 Dublin City University School of Nursing Psychotherapy and Community Health, Dublin, Ireland; 8 Mental Health Ireland, Dublin, Ireland; 9 Centre for Clinical Brain Sciences, The University of Edinburgh Division of Psychiatry, Edinburgh, Scotland, UK; 10 Trinity College Dublin Centre for Health Policy and Management, Dublin, Ireland; 11 University College Cork Department of Psychiatry and Neurobehavioral Science, Cork, Ireland

**Keywords:** public and patient involvement, capacity-strengthening, co-production, mental health, lived experience, participatory methods

## Abstract

**Background:**

Public and Patient Involvement (PPI) in mental health research is increasingly recognised as a moral and ethical imperative, necessary to increase the relevance and effectiveness of translation of research findings. Despite policy mandates and growing evidence of its benefits, PPI implementation in mental health research remains inconsistent. Little attention has been given to the state of scientific knowledge on PPI capacity strengthening in mental health research that can support more meaningful implementation. The aims of this scoping review are to: describe the content, implementation process, and theoretical underpinnings of PPI capacity-strengthening initiatives in mental health research; identify quantitative outcome measures and outcomes used to evaluate the initiatives’ impact on PPI contributors, research processes, and policy; and map barriers and enablers to the initiatives’ implementation.

**Methods:**

This scoping review will follow JBI and PRISMA-ScR guidelines. Sources will include peer-reviewed articles, grey literature, and organisational materials describing training or skill-building initiatives for adult PPI contributors in mental health research. Searches will be conducted in MEDLINE, Embase, PsycINFO, and CINAHL, supplemented by hand-searching, targeted internet searches, and stakeholder consultation. Data extraction will capture descriptive details, initiative content, outcomes, and contextual factors, with barriers and enablers categorised according to the Consolidated Framework for Implementation Research (CFIR) domains.

**Conclusion:**

This review will provide the first comprehensive synthesis of capacity-strengthening initiatives for PPI contributors in mental health research. Findings will inform the development of a co-designed blueprint for capacity-strengthening for PPI contributors, and progress broader efforts to embed lived experience expertise and general public perspectives equitably within mental health research systems.

## Background

Researchers and health systems are increasingly engaging people with lived experience of health conditions, their families/carers, and the public in research ( Health Service Executive (HSE), 2021; National Health Service (NHS), 2025; National Institute for Health and Care Research (NIHR), 2019; Vaughn & Jacquez, 2020). This is broadly referred to as ‘participatory research’, which emphasises conducting research *with* intended beneficiaries rather than *on* or *about* them ( Cornwall & Jewkes, 1995; NIHR, 2019; Vaughn & Jacquez, 2020). Frameworks such as participatory action research ( Baum *et al.*, 2006), citizen science ( Bonney *et al.*, 2009), and community-based participatory research ( Israel *et al.*, 2001; Wallerstein & Duran, 2006) share this principle of co-production.

A commonly used term in the healthcare and health systems context is ‘Public and Patient Involvement’ (PPI), which emerged from patient advocacy movements and now encompasses patients (also known as service users or lived experience contributors), families, healthcare professionals, policymakers, and the public ( Gilfoyle *et al.*, 2022; HSE, 2021). Often, research employing PPI will try to engage several of these stakeholders at once—and these identities are not mutually exclusive ( Gilfoyle *et al.*, 2022; HSE, 2021; NHS, 2025; Vaughn & Jacquez, 2020). Levels of involvement range from advisory roles to fully integrated research team membership, with responsibilities for shaping research questions, methodologies, and interpretation of data ( Bell *et al.*, 2023; Gallant *et al.*, 2025).

Over the last 10–15 years, PPI has been mandated in research both nationally, in Ireland, and internationally. National policies in the United Kingdom (UK) and Ireland, as well as prominent funding and publishing bodies, have required grant applicants to embed PPI in research funding applications. For instance, the UK’s NIHR and UK Research and Innovation (UKRI) have developed standards and resources to encourage PPI uptake ( NIHR, 2019; UKRI, 2025), while the Irish national health service, the Health Service Executive (HSE) and the PPI Ignite Network have developed similar resources that emphasise equity, accessibility, fair compensation, clear roles, and training for PPI contributors ( HSE, 2021; PPI Ignite Network, 2025). The Health Research Board and Research Ireland evaluate grant applications partly on how projects plan to enact PPI, in effect necessitating PPI in research proposals, Furthermore, journals such as the BMJ and the Lancet Psychiatry increasingly require statements about PPI usage as a condition for publication ( BMJ, 2018; Davis *et al.*, 2024). These developments reflect a growing recognition of lived experience expertise and general public perspectives as central to increasing the rigour, quality, and real-word relevance of health research. Beyond institutional and national mandates, PPI is also a moral and ethical imperative, helping to redress decades of epistemic injustice by valuing the knowledge of those with direct experience of health challenges ( Downs, 2025; McGorry *et al.*, 2024; Rose & Kalathil, 2019).

### Impact of PPI on contributors, research outcomes, and service provision

Evidence of the impact of PPI use in health research seems to be strongest at the project level. Several reviews on health and mental health care show that PPI: improves recruitment and retention ( Ennis & Wykes, 2013), enhances design and dissemination ( Allen *et al.*, 2020; Blackburn *et al.*, 2018; Mockford *et al.*, 2012), and empowers contributors by building confidence, ownership, and trust ( Brett *et al.*, 2014; Richmond *et al.*, 2023). PPI contributors have reported creating positive relationships with healthcare services after traumatic experiences in the mental health system, and developing new collaborative partnerships as a result of their participation in various aspects of the research cycle ( Allen *et al.*, 2020).

At the service provision level, some evidence suggests positive outcomes for mental health services. A review of nine studies by Ezaydi *et al.* (2023) found that co-produced mental health services were associated with increased attendance, treatment completion, fewer hospitalisations, and greater trust in the services. In-depth involvement of service users sometimes led to structural or cultural changes with potential long-term effects. However, such studies remain rare, and most focus on short-term outcomes.

At the policy and systems level, evidence is much weaker. A scoping review of healthcare research by Baumann *et al.* (2022) found that most studies describing and evaluating PPI assessed the PPI process itself rather than long-term policy impacts. Werner *et al.* (2025) similarly highlighted the lack of data on systemic impacts of PPI in psychiatric research, attributing this to limited standardisation of PPI impact measures and a lack of studies explicitly designed to evaluate long-term effects.

This gap in evidence may stem from the fundamental issue of low levels of PPI/co-production utilisation in mental health-related research, highlighted by the relatively small number of studies deemed eligible in reviews (e.g. Ezaydi *et al.*, 2023). In a scoping review by Gonzales and Jones (2024), only 2 out of the 23 studies that measured service user perspectives reported service user involvement in the actual study design or implementation. Furthermore, Chinsen *et al.* (2025)‘s systematic review of co-design in mental health interventions with young people found that their involvement was largely marginal.

### Challenges in implementing PPI in mental health research

Several reviews have identified key challenges in implementing PPI in mental health research, such as power inequities, tokenism, and stigma ( Biddle *et al.*, 2021; Brotherdale *et al.*, 2024; Ocloo *et al.*, 2021; Pinfold *et al.*, 2025). One of these challenges is the demand for narrow forms of evidence focused on standardised evaluation processes that simplify nuanced and heterogeneous experiences ( Armstrong & Byrom, 2025) and thus may be inadequate in measuring how each PPI contributor shaped the methods, results, and impact of a study. A lack of fully embedded approaches, together with inadequate use and reporting of evaluation measures, limits PPI’s potential to drive larger-scale changes in health systems and research processes ( Baumann *et al.*, 2022; Biddle *et al.*, 2021; Brotherdale *et al.*, 2024; Mockford *et al.*, 2012). Tokenism—superficial involvement that fails to provide decision-making opportunities or address power inequities ( Brotherdale *et al.*, 2024; Ocloo & Matthews, 2016; Ocloo *et al.*, 2021; Pinfold *et al.*, 2025; Richmond *et al.*, 2023)—is another common barrier, reinforcing unequal relationships between service users and researchers. Power imbalances impede participation by encouraging an ‘us vs. them’ mentality through labelling (‘PPI members’ vs. ‘researchers’), compartmentalising tasks, creating a false sense of decision-making power, discouraging critique of academic viewpoints, and limiting contact with senior researchers ( Allen *et al.*, 2020; Richmond *et al.*, 2023).

There are specific considerations for PPI in mental health research. For example, affective or cognitive effects of psychiatric medications may be brought to the forefront and impact PPI contribution in unforeseen ways. A systematic review linked sedatives such as benzodiazepines to impaired social cognition ( Haime *et al.*, 2021); echoing a meta-analysis associating them with deficits in working memory, processing speed, attention, and expressive language ( Crowe & Stranks, 2018). Other medications, including certain types of antipsychotics, have also been associated with cognitive impairment ( Haddad *et al.*, 2023; Joshi *et al.*, 2021). Such changes may influence contributors’ engagement, relationships, and preferred tasks in research projects. Navigating side effects can also form part of the “expertise” of PPI contributors when generating knowledge about relevant topics, such as the impact of iatrogenic harm ( Okkenhaug *et al.*, 2024).

Socio-emotional and cognitive effects arising from mental health difficulties also shape PPI contributors’ experiences. Low self-esteem, common both as a precursor to, and consequence of, mental health conditions, is linked to poorer wellbeing outcomes, while higher self-esteem is a protective factor ( de la Barrera *et al.*, 2022; Hilbert *et al.*, 2019; Keane & Loades, 2017; Rizwan & Ahmad, 2015). In PPI contexts, medication effects may converge with these socio-emotional and cognitive effects, producing varied impacts at individual, group, and project levels ( Allen *et al.*, 2020; Richmond *et al.*, 2023)—it is important for research teams to consider how these experiences can in fact provide unique insight into topics being studied.

Stigma further undermines participation, and researchers holding stigmatising beliefs about the abilities of people with mental health difficulties has been widely documented ( Allen *et al.*, 2020; Biddle *et al.*, 2021; Evans & Papoulias, 2020; Paul & Holt, 2017; Richmond *et al.*, 2023; Susanti *et al.*, 2020; Wadman *et al.*, 2019). While teams often support PPI in principle, they may emphasise the ‘risks’ of close collaboration. One study noted that “Poor health, especially mental health, was recognized as a significant barrier to effective co-production,” though some clinicians acknowledged the need for extra support ( Holland-Hart *et al.*, 2019, p. 96). Institutional processes can also deter participation, as illustrated by a lived experience research assistant who described being compelled to disclose highly sensitive personal information during Human Resource procedures, finding the process “distressing and re-traumatising” ( Richmond *et al.*, 2023, p. 224).

Another dimension of stigma is the privileging of academic knowledge over experiential knowledge, reinforcing the ‘us vs. them’ divide, and deepening epistemic injustice. Rose and Kalathil (2019) explore this issue within mental health research, arguing that within current academic contexts, “co-production between professionals and service users is fundamentally an unequal relationship” ( Rose & Kalathil, 2019, p. 8). Given the automatic privileging of academic or ‘rational’ forms of knowledge production in these settings, knowledge stemming from lived experience enters the field at a disadvantage. Paul and Holt (2017) observed that “The value placed on experiential knowledge vs. knowledge held within the research community emerged as a particularly complicated aspect of PPI in practice” ( Paul & Holt, 2017, p. 7). Addressing this injustice in the long term requires not just individual- or team-level strategies, but systemic reconstruction involving a re-evaluation of the very principles underpinning mental health research.

### Capacity-strengthening for PPI in mental health research

It is evident that unique barriers arise when implementing PPI in mental health research. Yet little attention has been given to the state of scientific knowledge on PPI capacity strengthening in mental health research that can address these challenges. Research capacity strengthening is defined by Centre for Capacity Research and Science for Africa Foundation (2024) as “enhancing the capacity of individuals and organisations to conduct, manage, share and apply research, while enabling national and sub-national research systems to effectively support both research and the linkages between research and practice.”. It is considered a long-term process that can be applied at the individual, institutional, or national level, and can be applied to improving the quality of PPI in mental health research. Several studies on PPI in research have highlighted the lack of, and need for, targeted training of PPI contributors, and point to skill-building activities ( Brett *et al.*, 2014; Holland-Hart *et al.*, 2019; Ocloo *et al.*, 2021; Opie *et al.*, 2023; Paul & Holt, 2017; Susanti *et al.*, 2020). An important gap in training for PPI contributors is in research literacy ( Allen *et al.*, 2020; Bedenik *et al.*, 2025). Tailored training for mental health research PPI contributors may help reduce tokenism, offer support in the ‘emotional work of using one’s lived experience identity, enhance skills sharing and support relationship building across research teams; and enhance comprehension and prevent overload from technical information ( Allen *et al.*, 2020; Capobianco *et al.*, 2023; Werner *et al.*, 2025). In a rapid review of training programmes for lived experience workforces, an analysis of 26 studies found that specialised, mental health-related training programmes resulted in improvements in confidence, communication skills, reflective listening, problem-solving, and overall improvements in job performance within the mental health services context ( Opie *et al.*, 2023). This points to the benefits that targeted capacity strengthening could bring to mental health research as well.

Moreover, implementation science provides tools to move PPI in mental health research beyond principles into practice by identifying the conditions that enable or hinder its meaningful adoption and sustainability. The Consolidated Framework for Implementation Research (CFIR; Damschroder *et al.*, 2022) was selected to guide this scoping review as it can help researchers examine how organisational culture, leadership, resources, and external policy drivers shape the extent to which PPI is embedded and valued within mental health research systems. The CFIR comprises five overarching ‘domains’ indicating the systemic level at which a barrier or enabler sits (i.e., the innovation design itself, the outer setting, the inner setting, the individual level, and the implementation process). Each of these domains also contains several sub-constructs (39 in total) into which barriers and enablers are categorised. This framework has been successfully adapted for social science and health research over the years, including in some recent scoping and systematic reviews in the area of mental health ( Garavito *et al.*, 2023; Higgins *et al.*, 2020; Le *et al.*, 2022; Mutschler *et al.*, 2022; Piat *et al.*, 2021; Rangachari *et al.*, 2022; Roshan *et al.*, 2025).

Considering that there is no standardised guidance on what the capacity strengthening of PPI contributors in mental health research should look like, this scoping review aims to address the objectives below.

## Objectives

The scoping review objectives are to:

1) Describe the content, underlying theoretical models/frameworks, and implementation processes of capacity-strengthening initiatives utilised to strengthen PPI in mental health research.
2) Describe any qualitative and quantitative outcome reported, including the measures used, to evaluate the experience or impact of the identified capacity-strengthening initiatives on lived experience contributors, the research process, and broader research or policy implementation goals.
3) Use the Consolidated Framework for Implementation Research (CFIR) to map identified barriers and enablers of implementing capacity-strengthening initiatives to CFIR’s 5 domains and 39 constructs.

The findings of the scoping review may additionally contribute to theory relating to PPI in mental health research by clarifying the concept of “capacity” in different contexts, identifying gaps in existing theory, and supporting the testing and refinement of theory going forward.

## Methodology

The scoping review protocol has been informed by the guidelines set forth by the Joanna Briggs Institute Manual for Evidence Synthesis ( Peters *et al.*, 2020) and the Preferred Reporting Items for Systematic Reviews and Meta-Analysis extension for Scoping Reviews (PRISMA-ScR) standards ( Tricco *et al.*, 2018). Some minor modifications to standard JBI protocol have been made in order to accommodate the specific needs of this review. The review is pre-registered with Open Science Framework ( https://osf.io/ndz67/).

This scoping review is the first step in a process evaluation of the enactment of PPI within a broader research programme entitled Vision To Action for Promoting Mental Health and Recovery – An Implementation Science Approach to “Sharing the Vision” (VISTA; https://vista-apro.eu/.) VISTA aims to develop comprehensive implementation blueprints to enact several recommendations from Ireland’s ‘Sharing the Vision’ mental health policy ( Department of Health, Ireland, 2021). The review findings will be applied to the PPI process currently being undertaken in VISTA, as well as to the PPI capacity-strengthening training blueprint that will be co-designed towards the end of the programme for eventual implementation in Recovery Colleges across Ireland. Recovery Colleges emphasise agency, self-direction, and personal growth, helping people understand and make sense of the experience of psychological distress and assisting them to learn and develop skills, confidence, and coping strategies. By providing transformative learning opportunities and a flexible, strengths-based, and empowering learning environment, service users, their supporters, and mental health professionals all learn from each other in these settings.

Additionally, this review is intended to benefit various stakeholders in the mental health research landscape. Researchers may find the review’s results helpful in improving the quality, inclusivity, and relevance of research through better PPI practices. PPI contributors can use the review’s findings to have more meaningful, supported roles, and gain skills and recognition in research settings. Research institutions and universities may use the findings to develop institutional structures (e.g., training, roles, governance) to sustain PPI. For funders and policy makers, findings can inform funding calls, shape national strategies, and identify requirements for impactful PPI in funded research. Moreover, the authors will use the review’s findings to produce policy briefings for the Health Services Executive (Ireland’s national health service) and the Department of Health regarding future directions for PPI in mental health research.

### PPI contribution

A PPI panel of seven members, ranging from people with lived experiences of mental health difficulties, family members/carers, and public representatives, has been involved in the development of this protocol, and will be regularly consulted for the data synthesis and write-up of the review. Lived experience PPI contributors have been involved in the VISTA programme from the grant-writing stage onwards; as such, they have been consulted on, and have shaped the following aspects of the review protocol: review aims and scope, inclusion/exclusion criteria, terminology used (e.g. ‘mental health challenges’, described below), and manuscript review/editing. The PPI panel will also be involved in the later stages of the review, such as in the stakeholder consultation phase of the grey literature search, data synthesis, and the write-up of the results manuscript.

The broader VISTA research programme is underpinned by this PPI panel, with each panelist embedded in one of the project’s work packages. As part of VISTA’s PPI process, before beginning to work together on research, the PPI panellists and academics underwent an induction phase involving capacity-strengthening activities such as team-building, crafting a personal narrative around recovery and mental health, consensus-building, influencing, and research literacy training.

### Eligibility criteria

Eligibility criteria for literature to be included is detailed in Table 1. The review uses a slightly modified version of the JBI’s PCC framework ( *participants, concept, context),* with the addition of *outcomes* as a final criterion to accommodate the review’s focus on barriers and enablers. In this review, *concept* will encompass any capacity-strengthening initiatives (such as training, webinars, educational materials, or workshops) that have been used with PPI contributors as the primary target in the research study.

**Table 1.** Eligibility criteria and operationalisation of the JBI’s PCC framework.

	Include	Exclude
**Participants**	• Adults who are PPI/lived experience contributors/general public representatives in mental health research (including peer researchers) • Research team members (academics) who may incidentally also receive capacity-strengthening training. • Studies with a mx of adult and youth PPI contributors will be included if adult/youth categories can be disaggregated, and if it can be determined that ≥50% of the contributors are adults.	• Adults who are only participants/data subjects in mental health research • Adults who are PPI/lived experience contributors in non-mental health research • Studies in which over 50% of PPI contributors are under 18 years of age. • People in ‘peer workforce’-type roles that are service-oriented, rather than focused on research contribution.
**Concept**	• PPI/lived experience/co-production within the research project at any stage of the research project (from pre-award stages, design, to dissemination) • Specific, purposeful capacity-strengthening initiatives focused on strengthening capacity to co-produce or engage with the research in mental health (examples of areas of capacity strengthening: teamwork, disclosure, confidence, public speaking, presentation, negotiation, ethics, personal development, professional development, values, principles) • Initiatives that target mixed groups alongside PPI contributors (e.g. clinicians, research, administrative staff, policy-makers) • Studies where PPI is one component of a broader co-production/implementation package	• Interventions that are not focused on capacity-strengthening for the purpose of co-producing research • Interventions that are only aimed at academic team members/researchers.
**Context**	• Study’s primary focus is on mental health (i.e. self-identified mental health challenges or clinical diagnoses of psychiatric conditions) • Studies in which mental health component is the dominant topic regardless of context (e.g. investigating depression in people with dementia) • Mental health research that incorporates PPI/lived experience insights in the research design and conduct	• Research that is not addressing mental health. In this review, dementia, intellectual disabilities, substance use is not defined as ‘mental health’ • Mental health research that does not incorporate any aspects of PPI or co-production
**Outcome**	• Barriers and enablers to implementation of capacity- strengthening initiatives • Evaluation of the PPI members’ experiences of the capacity- strengthening initiatives • Content of capacity-strengthening initiatives (setting, stage of research cycle, intended recipients, demographics of initiative participants, steps of initiative, etc) • Information on theoretical models or frameworks underlying the capacity-strengthening initiatives • Information on any qualitative and quantitative implementation or impact outcomes that the initiatives might measure, across PPI contributor, project, service, and system levels	• Barriers and enablers that are not related to specific capacity- strengthening initiatives for PPI members in mental health (e.g. barriers/enablers to convincing researchers to take up PPI in their research)
**Types of Data Sources**	• Published, peer-reviewed academic journal articles • Journal opinion pieces and editorials • Grey literature (key organisation websites and related materials, additional resources from key stakeholders, unpublished theses, journal editorials) • Articles published in English	• Evidence syntheses articles (e.g. scoping, systematic, umbrella reviews, meta-analyses) • Conference proceedings, papers, and presentations. • Any protocol papers • Articles not published in English

Literature and resources dealing with mental health research will be considered; these sources must primarily have a focus on mental health, (there can be a secondary or incidental focus on other topics such as physical health), and must pertain to adults who are lived experience PPI contributors. Studies in which over 50% of PPI contributors are under 18 years of age will be excluded. Additionally, sources must also describe, to a meaningful extent, the upskilling, training, or capacity-strengthening of PPI contributors, and this capacity-strengthening should be aimed at enhancing the engagement of the PPI contributors in research projects (e.g. research literacy modules/manuals, ethics and data protection training, public speaking and presentation skills). The literature must describe the content of the capacity-strengthening initiative, and may additionally describe its implementation process and any theoretical underpinnings of the initiative. Studies focused on any stage of the research cycle (pre-award stages—post-publication, dissemination stages) will be considered. Lastly, we will consider literature that indicates, implicitly or explicitly, barriers and enablers relating to the implementation of these capacity-strengthening initiatives. We will not consider: sources that do not have a mental health focus; sources that do not pertain to lived experience PPI members; sources that do not describe any capacity-strengthening initiatives; sources whose capacity-strengthening attempts are not at least partially targeted towards PPI contributors; sources whose upskilling/capacity-strengthening is not aimed at the enhancement of co-production capabilities; and sources that do not mention or allude to barriers or enablers relating specifically to the capacity-strengthening initiatives.

The review will screen published and unpublished literature that addresses the review’s aims, and will include quantitative, qualitative, and mixed methods designs. Systematic reviews, scoping reviews, umbrella reviews, and meta-analyses will be excluded; however, their reference lists will be manually checked for relevant primary sources. Conference proceedings, papers, and presentations will be excluded. A grey literature strategy (described below) will also be used, as it is expected that much of the relevant details on specific training for PPI contributors and research teams will be found in guidelines, training materials, and other community-based and non-peer reviewed sources.

The scope of what the review would consider under mental health research, as well as the preferred terminology to use in this area, was decided on in consultation with VISTA’s lived experience PPI team members. The phrase ‘mental health difficulty’ was seen by both researchers and lived experiences contributors as the most comprehensive and least stigmatising term to use through this review and is the term used in the ‘Sharing the Vision’ ( Department of Health, 2021) policy. We will consider research dealing with any of the mental and behavioural difficulties outlined in the International Classification of Diseases—11 ^th^ Revision (ICD-11) ( World Health Organisation, 2022).

Search strategy & information sourcesThe concepts comprising the search strategy include: PPI/Lived Experience, Mental Health, and Specific Capacity-Strengthening Initiatives. After the research team compiled lists of terms found in the literature for each concept, the study librarian selected the most relevant terms to include in the search string (outlined in Table 2)—this search string was then modified for the following databases: MEDLINE (EBSCOhost), Embase (Elsevier), PsycINFO (EBSCOhost), and CINAHL (EBSCOhost). The search does not have language, geographical, or time limits, in order to obtain all relevant studies, however only studies published in English will be included in the review. The search will be open to articles from inception until the search date. Before commencing the data extraction stage, one of the researchers (SAV) will hand-search the reference lists of included studies to check for any additional studies that may meet the inclusion criteria.

**Table 2.** Search strategy used.

	General terms	Search Strategy for Databases
**Concept 1: Public and Patient Involvement/Lived Experience/Co-Production **	Lived experience, consumer, service user, patient, public, family, carer (either/or), PPI	(((consumer* OR famil* OR patient* OR public OR “service user*”) NEAR/1 (involv* OR “lived experience*”)) OR “co-research*” OR “co-produc*” OR coresearch* OR coproduc* OR “PPI”)
		AND
**Concept 2: Mental Health**	Specific mental health difficulties/diagnoses as guided by the ICD-11	(anxiet* OR anxious OR depress* OR “eating disorder*” OR ((mental*) NEAR/2 (disorder* OR health* OR ill*)) OR “mood disorder*” OR psychiatric OR psychosis OR psychotic OR schizophreni* OR “self harm*” OR suicid* OR bipolar* OR obsessive* OR traumatic* OR personality* OR substance* OR dissociative*)
		AND
**Concept 3: Specific capacity-building/strengthening initiatives used in the study**	Capacity building, capacity strengthening, toolkit, education, training, learning, initiative, competency building/strengthening, upskill, skill building, supports	(((capacity OR competency OR skill*) NEAR/1 (build* OR strengthen*)) OR education* OR initiative* OR learning OR support* OR training OR toolkit* OR upskill*)

The database search was conducted on November 7 ^th^, 2025.

Grey literature will be obtained through multiple avenues. Firstly, websites of key national and international PPI-related community, healthcare, policy, and research organisations, and related materials such as guidelines or standards published by them, will be assessed for relevant information. Examples of such organisations are PPI Ignite, INVOLVE, the McPin Foundation, Mental Health Ireland, websites of recovery colleges, PPI-related web pages of national health systems such as the HSE and NHS, and blogs of lived experience PPI contributors and researchers. Web repositories such as Overton (for policy documents), WHO IRIS (World Health Organisation repository), EU Publications (for EU law and policy), and UN Library will be consulted. Secondly, sources such as unpublished theses (via open university repositories) will be considered. Lastly, key stakeholders working in the PPI space (such as prominent lived experience/PPI advocates, lived experience researchers, experts in the field of co-production, and other key community members) will be contacted directly to source additional, potentially unpublished resources on capacity-strengthening.

Stakeholder consultation will occur concurrently with the grey literature search, once title and abstract screening are completed. Firstly, subject matter experts are part of the review team; as such, they will direct the review team to important and relevant initiatives that the grey literature search has not covered. Going beyond the review team itself, next, a list of relevant subject matter experts internationally (with particular focus on those familiar with the Irish, EU, and UK research systems) will be created. Stakeholders will be contacted via email, with a specific request to direct the authors towards any relevant capacity-strengthening materials (published or unpublished initiatives, policies, evaluations, etc.) for PPI contributors in the mental health space that they are aware of. Those who do not reply initially will be contacted one more time, after which the authors will no longer attempt contact. The additional material gathered through this consultation will be screened against the review’s inclusion and exclusion criteria.

The grey literature search will be documented and reported as part of the PRISMA flowchart in the final manuscript. Documentation will include, where applicable, the name of the website where the source was found, the source’s URL, date of the search, search terms used, number of results, other information on how the search was conducted, and reason for exclusion of the source.

### Screening

Database search results will be uploaded onto Covidence ( Covidence systematic review software, 2025), where duplicates will be removed, and further screening will take place. At the title and abstract level, all references will be screened by two independent reviewers. Full texts will also be reviewed independently by two reviewers, and reasons for excluding articles will be recorded and used in the PRISMA flow chart. At each of these stages, screening will first be pilot-tested on 20% of the studies before continuing; conflicts between reviewers will then be closely monitored, and regular conflict resolution meetings between the reviewers will take place to resolve them—if needed, a third person from the team will be invited to resolve the conflict. If a significant number of conflicts arise during these first two stages, a further 10% of articles will undergo pilot-testing. Regular meetings will be held with the review team to ensure that there is a standardised approach to screening.

### Data extraction

Data extraction will occur via the extraction function on Covidence; once data is initially extracted there, it will be transferred to NVivo ( Lumivero, 2024), and if needed, on Microsoft Excel (Microsoft Corporation, 2025). Here, additional descriptive and thematic analyses will be performed. The JBI data extraction template has been modified to address review objectives 1 and 2 (Appendix A). The template will capture the studies’ descriptive information (e.g., authors and year of publication, country, study design, aim), population characteristics (e.g., sample size and type, age range, and description of the research team) details of the intervention (e.g., details of the capacity-strengthening initiatives (setting, stage of research cycle, intended recipients, demographics of initiative participants, steps of initiative, etc.), theoretical models/frameworks underpinning the initiatives, outcomes of the initiatives, gaps in training identified, any PPI monitoring or evaluation tools used), context (e.g., mental health area/difficulty, challenges identified relating to mental health needs of PPI contributors). This set of data addresses objectives one and two of the review.

Data relevant to the review’s third objective (identifying barriers and enablers) will be collected in line with CFIR; Appendix C; Damschroder *et al.*, 2022). This review will structure significant information relating to the implementation of PPI capacity-strengthening initiatives according to the 39 constructs of CFIR, thereby extracting specific pieces of data that can be more easily translated for use in future implementation projects. The CFIR domains will be operationalised using the CFIR research mapping tool developed by Damschroder *et al.* (2022), and data will be coded to the framework using guidance from the CFIR Construct Coding Guidelines ( Damschroder *et al.*, 2022).

The data extraction template (Appendix A) will first be pilot tested on 20% of studies by two independent reviewers before being used for the remaining studies, with regular discussions between the reviewers to resolve any problems with the template or major differences in the extracted data. In case the template must be revised, the amended template will be reflected in the published scoping review. It is possible that there will be some conceptual overlap between data extracted for objectives one and two, and data extracted for objective three via the CFIR domains.

### Analysis and presentation of findings

Data synthesis will occur in NVivo (and Excel as needed). Scoping review findings will be presented in line with JBI and PRISMA-ScR guidelines ( Peters *et al.*, 2020; Pollock *et al.*, 2022; Tricco *et al.*, 2018). A descriptive narrative account of the existing quantitative, qualitative, and mixed methods literature will be provided; augmented by tables that provide an overview of included study characteristics and methodologies used. Findings will focus on mapping, summarising, and charting available evidence; identifying knowledge gaps; clarifying concepts; and reviewing research conduct. This account will clarify how the scoping review results relate to, and address, its research aims. To achieve this, for aim 1 (capacity-strengthening initiative content and implementation processes) and aim 2 (outcome measures), a basic descriptive analysis will be conducted. For aim 3 (implementation barriers and enablers), data will be analysed deductively by mapping individual study findings to the CFIR.

### Limitations

While the review takes steps to achieve comprehensiveness, some limitations still arise. Firstly, as this is a scoping review (rather than a systematic review), quality and risk-of-bias assessments will not be conducted, which may limit the review’s ability to provide actionable recommendations. Additionally, we expect a large portion of the literature that will be analysed will be derived from non-peer reviewed or unpublished sources such as websites and documentation of community-level initiatives. Combined with the restriction to English-only literature (which may underrepresent non-English speaking and low- and middle-income contexts in which PPI and capacity-strengthening are growing), these limitations will reduce the extent to which the review’s findings can be used as an evidence base for policy or guidelines internationally and their transferability to diverse research contexts. Furthermore, given the decentralised nature of the non-peer reviewed sources, and the review’s use of subject matter experts in identifying grey literature, some relevant resources may be missed in the literature search, particularly those that may not yet be published. Lastly, as PPI practices and guidelines evolve at a fast pace, there is a risk that the review could miss out on very recent initiatives or approaches by the time it is completed. Despite the limitations, this review will mark the first time that capacity-strengthening for PPI in mental health research contexts has been examined in a systematic way, and will springboard to support PPI capacity-strengthening both within and beyond the VISTA programme.

## Conclusion

This scoping review aims to gather and consolidate existing information on capacity-strengthening initiatives for PPI contributors in mental health research, identifying barriers and enablers to implementing these initiatives within research contexts. To do this, the review grounds itself in an implementation science-based approach, utilising a detailed framework, the CFIR, to extract and analyse data related to barriers, enablers, and outcomes of capacity-strengthening initiatives. This approach will allow the research team to more effectively comment on how relevant domains of research settings can be modified to reduce some of the major challenges and inequities of PPI.

### Ethics and consent

Ethical approval and consent were not required for this scoping review.

## Data availability

### Underlying data

No data are associated with this article.

### Extended data

This protocol is preregistered on Open Science Framework: “Content, implementation, and evaluation of capacity-strengthening initiatives for public and patient involvement in mental health research: a scoping review protocol informed by implementation science” ( Anand-Vembar *et al.*, 2025) https://doi.org/10.17605/OSF.IO/EYHGM

This project contains the following extended data:

1) Supplementary File 1:

Manuscript 14287 Appendices ( https://osf.io/eyhgm/files/zkbm9).

2) Supplementary File 2:

Manuscript 14287 PCC Chart and Search Strategy Table ( https://osf.io/eyhgm/files/76gzd).

Data are available under the terms of the Creative Commons Zero “No rights reserved” data waiver (CC0 1.0 Universal).
