# Supplementary material for: Content, implementation, and evaluation of capacity-strengthening initiatives for public and patient involvement in mental health research: a scoping review protocol informed by implementation science
Source: HRB Open Res. 2026 May 7;8:123. Originally published 2025 Nov 18. [Version 2] doi: 10.12688/hrbopenres.14287.2 (PMC12820475; doi:10.12688/hrbopenres.14287.2)
Supplement: Peer review history

# Reviewer report: Reviewer response for version 2

**DOI:** 10.21956/hrbopenres.15883.r55202

**Published:** 2026-05-22

**Authors:** Elizabeth Morrow

**Affiliations:** 1 Royal College of Surgeons in Ireland, Dublin, Ireland

**Reviewer 1. Response to Author’s revisions (version 2)**

 Thank you to the authors for their careful and constructive revisions made in response to our comments. The manuscript has been substantially strengthened, particularly in relation to clarification of aims and objectives, stakeholder consultation processes, PPI contributor involvement, methodological transparency, dissemination considerations, and acknowledgement of review limitations. The revisions improve the coherence and overall rigor of the protocol and better situate the review within the existing mental health PPI literature.

 Most reviewer comments have now been addressed satisfactorily. However, there remain a small number of conceptual and methodological areas that would benefit from further clarification prior to final acceptance. We recommend consideration of the following comments to strengthen the manuscript.

 1. Theoretical grounding of PPI and involvement

 While the authors have clarified that theoretical frameworks underpinning initiatives will be extracted during the review process, the protocol itself would still benefit from a slightly stronger engagement with the broader theoretical literature on PPI and participation. The original concern was not necessarily that a single theory should structure the review, but that the protocol could more clearly acknowledge its relationship to wider traditions of participation, co-production, and involvement scholarship (for example, participation continuums, democratic participation traditions, or models of power-sharing in involvement). A brief contextual paragraph would help strengthen the conceptual framing of the review in existing theory.

 2. Justification for selection of CFIR

 The additional clarification regarding CFIR is helpful. However, the rationale for selecting CFIR over other implementation science frameworks could still be articulated explicitly, if only briefly. This does not require an extensive comparison with all alternative frameworks, but a concise justification explaining why CFIR is particularly appropriate for analysing implementation barriers and enablers in heterogeneous PPI capacity-strengthening initiatives would improve methodological transparency.

 3. Inclusion of user-led and survivor research traditions

 The clarification regarding search strategy sensitivity is useful; however, it remains unclear whether survivor-led, advocacy-led, or user-led research traditions are conceptually included within the scope of the review. Given the significance and influence of these traditions within mental health research and PPI scholarship, an explicit statement regarding their inclusion/exclusion would improve clarity and reproducibility. It is possibly an area that the PPI contributors to the study will want clarity on too and whether this evidence is to be included.

 These remaining points are relatively minor and do not detract from the overall quality and importance of the protocol or the potential contribution of this research to theory and practice. Subject to these further clarifications, we believe the manuscript is suitable for acceptance.

Is the study design appropriate for the research question?

Yes

Is the rationale for, and objectives of, the study clearly described?

Partly

Are sufficient details of the methods provided to allow replication by others?

Partly

Are the datasets clearly presented in a useable and accessible format?

Not applicable

Reviewer Expertise:

NA

I confirm that I have read this submission and believe that I have an appropriate level of expertise to confirm that it is of an acceptable scientific standard, however I have significant reservations, as outlined above.

# Reviewer report: Reviewer response for version 2

**DOI:** 10.21956/hrbopenres.15883.r55200

**Published:** 2026-05-12

**Authors:** Miriam Stanyon

**Affiliations:** 1 Derbyshire Healthcare NHS Foundation Trust, Derby, UK

Thank you for making the changes suggested. Your report of changes was thorough and well executed. I approve this article on a very important topic, for indexing and all the best with the review proper.

Is the study design appropriate for the research question?

Yes

Is the rationale for, and objectives of, the study clearly described?

Yes

Are sufficient details of the methods provided to allow replication by others?

Partly

Are the datasets clearly presented in a useable and accessible format?

Not applicable

Reviewer Expertise:

PPI in the area of mental health education and research, systematic reviewing, JBI synthesis methods

I confirm that I have read this submission and believe that I have an appropriate level of expertise to confirm that it is of an acceptable scientific standard.

# Reviewer report: Reviewer response for version 1

**DOI:** 10.21956/hrbopenres.15719.r52834

**Published:** 2026-01-20

**Authors:** Ramesh Kumar Sangwan

**Affiliations:** 1 Indian Council of Medical Research National Institute for Implementation Research on Non-Communicable Diseases, Jodhpur, India

- The protocol is **conceptually strong**, rooted in participatory research, epistemic justice, and implementation science, and clearly justified as the first systematic mapping of capacity-strengthening initiatives for PPI in mental health research.​
- The aims, review questions, and methods are generally coherent and appropriate for a scoping review, with suitable use of JBI methodology, PRISMA-ScR, and CFIR to structure extraction and analysis.​
- The major areas needing refinement relate to: operational clarity (PPI definition and boundaries), search/eligibility precision (especially grey literature and non‑English work), outcome/CFIR operationalisation, and tightening some sections for readability.
- **Strong rationale and positioning**
  - Background provides an excellent synthesis of the evolution of PPI, its moral/ethical basis, and specific complexities in mental health (medications, stigma, epistemic injustice, tokenism, power).​
  - Clear articulation that the focus is on *capacity-strengthening for PPI contributors* rather than generic PPI or generic training, and that this is a neglected area despite policy mandates.​
- **Clear aims and review questions**
  - Aims are explicit: mapping state of knowledge, identifying outcome measures, and mapping barriers/enablers using CFIR.​
  - The link between aims and the intended use (VISTA programme, blueprint for Recovery Colleges) is well stated and increases policy/practice relevance.​
- **Methodological robustness**
  - Use of JBI Manual and PRISMA-ScR, with explicit description of how reporting will follow these.​
  - Use of CFIR to structure barriers and enablers, including reference to the updated 2022 articulation and 39 constructs.​
  - Planned dual pilot of data extraction on 20% of studies with iterative refinement and consensus processes.​
- **Thoughtful handling of PPI within the review itself**
  - PPI panel composition, roles, and induction/capacity-strengthening activities are clearly described, showing meaningful engagement rather than tokenism.​
  - Positioning the protocol as part of a broader process evaluation of PPI implementation within VISTA is a notable strength.​
- Main weaknesses / risks
- **Scope and definitions need sharper boundaries**
  - “Mental health research”, “lived experience PPI contributors”, and “capacity-strengthening initiatives” are described, but practical boundaries could be clearer (e.g. interventions that target mixed groups including clinicians; studies where PPI is one component of a broader co‑production/implementation package).​
  - The exclusion of studies where >50% of contributors are under 18 is stated, but the handling of mixed adult–youth samples (e.g. 30–40% under 18) is not fully clarified; this could lead to inconsistent decisions.​
- **Outcome conceptualisation is under-specified**
  - While the protocol aims to “identify outcome measures used to evaluate the impact of capacity-strengthening initiatives”, there is no explicit categorisation of outcomes (e.g. at contributor, project, service, and system levels) or how purely process-oriented data (e.g. satisfaction, feasibility) will be treated.​
  - Some outcomes overlap conceptually with CFIR constructs (e.g. self-efficacy, readiness), which may complicate coding unless this is pre‑planned.​
- **Grey literature strategy could be more explicit and reproducible**
  - The protocol acknowledges that much of the relevant material will be in guidelines, training materials, and organisational documents, but the search procedures (databases, key websites, search strings, use of expert networks) remain high-level.​
  - Reliance on subject matter experts to identify resources is appropriate but risks selection bias without a more structured description of how they will be engaged and how saturation will be judged.​
- **Language and geographical limitations**
  - Restricting to English-only sources is acknowledged as a limitation but may systematically underrepresent non‑Anglophone contexts where PPI and capacity-strengthening are growing (e.g. Latin America, Asia, francophone Europe).​
  - The protocol might be perceived as heavily UK/Ireland-centric given the policy examples and VISTA context, which may limit transferability if not explicitly addressed in discussion/interpretation.​
- Specific, actionable suggestions
  1. Clarify scope and operational definitions
- Add an operational definition box/table early in the methods with:
  - **PPI contributor**: clarify inclusion of carers, public representatives, peer workers, and whether “peer workforce” roles employed by services are in or out of scope.​
  - **Mental health research**: specify inclusion/exclusion of adjacent areas (e.g. substance use, neurodevelopmental conditions, dementia, psychosocial disabilities).​
  - **Capacity-strengthening initiative**: make explicit whether you will include:
    - Training embedded in broader implementation packages.
    - One-off workshops versus longitudinal programmes.
    - Interventions aimed at both PPI contributors and researchers if substantial content targets PPI contributors.​
- For age criteria, add a sentence on how you will treat studies with mixed adult–youth PPI (e.g. include if ≥50% are adults or if data for adults can be meaningfully disaggregated).​
- 2. Tighten eligibility criteria and PCC+O description
- In the eligibility section, consider presenting the PCC+O framework as a compact table (participants, concept, context, outcomes) with precise operationalisation and examples of borderline cases.​
- Under “Outcomes”, specify:
  - Which types of outcome will be extracted (e.g. changes in research literacy, confidence, role clarity, engagement, co‑production quality, project outputs, service changes).​
  - How purely descriptive or narrative reflections (common in grey literature) will be treated and coded.​
- 3. Strengthen the search strategy description
- Explicitly list:
  - Databases (already present: MEDLINE, Embase, PsycINFO, CINAHL) plus time frame (from inception to a specified date).​
  - Example search string (PPI terms × mental health × capacity-building/training × research), either in the main text or as an appendix.​
- For grey literature, specify:
  - Key portal sites (e.g. NIHR, INVOLVE/NIHR, NHS England, HSE, PPI Ignite Network, mental health NGOs, Recovery Colleges, WHO, etc.).​
  - How web search engines will be used (e.g. first 3–5 pages of results for defined search strings; date filtering; language limits).​
  - How you will document and report grey literature sources (e.g. in a PRISMA-ScR flow diagram and supplementary tables).​
- 4. Refine data extraction and CFIR mapping plan
- Expand one short paragraph to clarify:
  - How CFIR constructs will be operationalised for this topic (e.g. pre‑defined coding framework based on Damschroder 2022; allowance for inductive sub-codes within constructs).​
  - How you will handle data that does not fit CFIR well, including whether an additional inductive thematic layer will be applied.​
- For outcome measures (aim 2), specify that you will:
  - Distinguish between validated instruments, ad hoc measures, and qualitative outcome indicators.
  - Capture psychometric properties or at least basic details when available (e.g. scale names, domains, scoring) to inform future evaluation design.​
- 5. Reporting and limitations
- In the limitations section, consider briefly adding:
  - A note on the potential under‑representation of low- and middle-income country contexts due to language and grey literature access constraints.​
  - A short statement about how findings will be framed as hypothesis‑generating and agenda‑setting rather than as a definitive causal evidence base, in line with the scoping review design.​
- Language and structure refinements
- The manuscript is generally well written, but some paragraphs in the background are very dense and could be broken into shorter units to improve readability for non‑specialist readers.​
- Where long citation clusters occur (e.g. lines with 5–7 references), consider checking whether all are needed or whether a smaller set of exemplar references would suffice.​
- Check for minor formatting consistency (e.g. hyphenation of co‑production vs co-production, use of em dashes, and consistency in terms like capacity-strengthening vs capacity building).

Is the study design appropriate for the research question?

Yes

Is the rationale for, and objectives of, the study clearly described?

Yes

Are sufficient details of the methods provided to allow replication by others?

Yes

Are the datasets clearly presented in a useable and accessible format?

Yes

Reviewer Expertise:

Adolescent Health, Maternal & Child Health, Mental Health and Medical Sociology.

I confirm that I have read this submission and believe that I have an appropriate level of expertise to confirm that it is of an acceptable scientific standard, however I have significant reservations, as outlined above.

# Response

**Published:** 2026-04-07

**Authors:** Shaakya Anand-Vembar

**Affiliations:** Trinity College Dublin School of Nursing and Midwifery, Dublin, Ireland

Dear Dr Ramesh Kumar Sangwan, 

 We thank you for taking the time to peer review our scoping review protocol and greatly appreciate your expertise and suggestions. Your comments have provided us with important insights that have strengthened our understanding of the protocol's scope and inclusion criteria, allowed us to develop a more detailed grey literature strategy, and helped us improve the conceptualisation of our outcomes.  

 We have carefully considered the specific points raised in the review, and have addressed them on a point-by-point basis in this document: https://docs.google.com/document/d/1laCzkT8bjSUagmVchmRTrH1ECNonIZ9x/edit?usp=sharing&ouid=107131347417362362861&rtpof=true&sd=true

 Please refer to both the tracked changes in the revised manuscript, as well as tracked changes in the appendix file. Changes pertaining to your comments have been marked as 'Reviewer 3 Comment __’ in the comments within the revised manuscript, for ease of tracking. 

 We hope that the revisions sufficiently address your comments; any suggestions that were found to be out of scope for this review will certainly be considered in the write-up of the final publication.  

 Thank you again for your time and attention, 

 Dr Shaakya Anand-Vembar, on behalf of all authors

# Reviewer report: Reviewer response for version 1

**DOI:** 10.21956/hrbopenres.15719.r52835

**Published:** 2026-01-20

**Authors:** Miriam Stanyon

**Affiliations:** 1 Derbyshire Healthcare NHS Foundation Trust, Derby, UK

This article reports a protocol for a scoping review following JBI guidelines to look for ways to build capacity for involvement of patients and members of the public in mental health research.

 Barriers and enablers will be categorised according to the CFIR domains.

 The methodology for searching and screening is all thorough and well explained. My one comment for data extraction is that your data extraction method for the third aim is difficult to conceptualise. Maybe in your appendix you could include an example, with example text that would come under the different constructs, or state the kind of elements from an article that may be extracted into the table.

 Other than this I think this is a very well written protocol.

Is the study design appropriate for the research question?

Yes

Is the rationale for, and objectives of, the study clearly described?

Yes

Are sufficient details of the methods provided to allow replication by others?

Partly

Are the datasets clearly presented in a useable and accessible format?

Not applicable

Reviewer Expertise:

PPI in the area of mental health education and research, systematic reviewing, JBI synthesis methods

I confirm that I have read this submission and believe that I have an appropriate level of expertise to confirm that it is of an acceptable scientific standard.

# Response

**Published:** 2026-04-07

**Authors:** Shaakya Anand-Vembar

**Affiliations:** Trinity College Dublin School of Nursing and Midwifery, Dublin, Ireland

April 7th, 2026

 Dear Dr Miriam Stanyon, 

 We thank you for taking the time to peer review our scoping review protocol; we greatly appreciate your expertise and your comments on the protocol.  

 Your suggestion regarding the data extraction tool has helped us increase the review's methodological robustness. Please see below for our response to your comment, and please refer to changes in the appendix file, in Appendix A, for the changes pertaining to your comment. 

 We hope that the revisions to the data extraction tool sufficiently address your suggestion. 

 Thank you again for your time and attention, 

 Dr Shaakya Anand-Vembar, on behalf of all authors 

 Reviewer 2 Comment: 

 My one comment for data extraction is that your data extraction method for the third aim is difficult to conceptualise. Maybe in your appendix you could include an example, with example text that would come under the different constructs, or state the kind of elements from an article that may be extracted into the table. 

**Author Response:** Example text for each domain has been added to Appendix A (Data Extraction Tool Informed by CFIR).

# Reviewer report: Reviewer response for version 1

**DOI:** 10.21956/hrbopenres.15719.r51895

**Published:** 2025-12-30

**Authors:** Elizabeth Morrow, Helen Sloan, Llinos Haf Spencer, Mary Lynch

**Affiliations:** 1 Royal College of Surgeons in Ireland, Dublin, Ireland; 2 Bournemouth University, Poole, England, UK; 3 Faculty of Nursing and Midwifery, Royal College of Surgeons in Ireland (RCSI) University of Medicine and Health Sciences, Ireland, Ireland

**INTRODUCTION**

 Our review of the article by Anand-Vembar and co-authors is intended to complement, enhance, and constructively support this important scoping review by critically engaging with its aims, methodological approach, and conceptual framing. As the authors state, public and patient involvement (PPI) has become an increasingly important component of mental health research, reflecting a broader shift towards participatory, inclusive, and ethically grounded health research practices. It is important to foreground the scoping review in the realist questions - what is the evidence on what works, for whom, when, and why, and who may currently be missing out?

 There is a growing body of evidence to show that meaningful PPI has the potential to enhance the relevance, integrity, acceptability, quality, and impact of research by involving those with firsthand experience or ‘lived experience’ throughout the research process. Additionally, benefits associated with involvement extend beyond improved research relevance and quality to include opportunities for mutual learning between researchers and public contributors, the development of shared understanding through the integration of experiential and academic knowledge, and the redistribution of decision-making power, thereby challenging traditional hierarchies within mental health research and promoting more equitable and collaborative research practices. Thus, PPI can be understood through several conceptual lenses. It may be instrumental, where involvement is used to improve the quality or efficiency of research; normative or democratic, where involvement is valued as an ethical right and a matter of accountability; and substantive, where public contributors’ lived experience shapes and influences research questions, methods, or outcomes.

 Despite strong policy support and growing expectations from funders and institutions, the implementation of PPI in mental health research remains inconsistent, and researchers often report challenges related to skills, resources, organisational support, and sustainability. Capacity-strengthening initiatives have therefore emerged as a key strategy to support researchers, public contributors, and organisations in developing the knowledge, confidence, and structures required for effective involvement.

**Article summary**

 The article by Anand-Vembar et al. presents a study protocol for a scoping review that aims to identify and synthesise evidence on the barriers and enablers to implementing capacity-strengthening initiatives for PPI in mental health research. Guided by the Consolidated Framework for Implementation Research (CFIR), the protocol outlines a systematic and theory-informed approach to mapping existing initiatives and the contextual factors influencing their implementation. As a study protocol published in an open-research format, the article does not report findings but instead provides transparency around the planned methods and analytical framework. This review critically examines the article’s rationale, methodological approach, and anticipated contribution to the field, considering its strengths, limitations, and potential value for advancing PPI practice and implementation science in mental health research.

**Focus of the review**

 In the study protocol, capacity-building is defined as initiatives designed to enhance the knowledge, skills, confidence, and resources of both researchers and public contributors, as well as the organisations that support them, to enable meaningful public and patient involvement (PPI) in mental health research. The concept encompasses both individual-level capacity, such as training and mentoring, and organisational-level capacity, including the development of supportive structures, processes, and cultures for involvement. Importantly, capacity-building is framed not merely as increasing the number of involvement activities but as fostering sustainable, effective engagement. By situating capacity-building within the Consolidated Framework for Implementation Research (CFIR), the protocol positions it within a broader system of enablers and barriers, highlighting the interplay between people, practices, and organisational contexts in shaping successful PPI implementation.

**GENERAL COMMENTS**

 The study protocol by Anand-Vembar et al. presents a rigorous and transparent plan for a scoping review of capacity-strengthening initiatives for public and patient involvement (PPI) in mental health research. By explicitly mapping barriers and enablers using the Consolidated Framework for Implementation Research (CFIR), the protocol demonstrates a strong theoretical grounding and methodological clarity. While it does not report empirical findings, the detailed approach enhances reproducibility and provides a clear roadmap for subsequent analysis. Moreover, the open-access publication and adherence to open-research principles align with current best practices in research transparency and knowledge sharing, which is particularly relevant for advancing evidence-informed PPI in mental health.

 Overall strengths of the study protocol are:

- Transparent methodology: The article clearly outlines the databases, inclusion criteria, and data extraction processes.
- Focus on capacity-building: Addresses an important gap in PPI research by examining how initiatives are implemented, not just whether they exist.
- Theoretical framework: Uses CFIR to structure barriers and enablers, providing a robust implementation science lens for the analysis.
- Open-access publication: Promotes transparency, reproducibility, and early community engagement in the scoping review.

Main limitations:

- Justification could be stronger: The rationale for the review could be strengthened by engaging more with existing literature bases, theory and PPI frameworks.
- Potentially narrow scope: The review may miss innovative, user-led or informal PPI initiatives not captured in published or indexed literature. Grey literature and stakeholder consultation phases of the review could support a more robust process.
- Limited discussion of theory and context variability of PPI across international settings
- CFIR is a suitably broad framework, but alternative implementation frameworks are not discussed and differences across countries, health systems, or cultural contexts may not be fully addressed.
- Unclear stakeholder involvement in review process: While the review is about PPI, the protocol does not detail whether public contributors were involved in shaping the review question, or exactly how PPI members will be involved in the analysis or interpretation of the scoping review results.
- Rapidly evolving field: PPI practices and guidelines change quickly; there is a risk the review could miss very recent initiatives or emerging approaches by the time it is completed.
- Data synthesis challenges: Scoping reviews often map evidence without assessing quality in depth, which may limit actionable recommendations.

**SPECIFIC COMMENTS**

**Attention to the drivers of PPI in research:**

 The paper does not explicitly acknowledge key drivers of PPI in research, such as policy mandates, high-profile failures within health systems (for example, Bristol Royal Infirmary or Alder Hey), or the advocacy and lobbying roles of consumer, survivor and service user groups. Nor does it clearly link the discussion of involvement to influential policy developments and inquiries, such as the Francis Inquiry, which have been instrumental in shaping contemporary expectations around patient involvement in healthcare and health research. The absence of this contextual and policy grounding risks presenting involvement as an abstract or optional activity, driven primarily by anticipated outcomes, rather than as a practice deeply rooted in historical lessons from system failures, ethical imperatives, research governance and regulatory requirements.

**Existing research and theory:**

 The authors touch only briefly on several important issues- such as power imbalances, stigma, and limited capacity to contribute to research- each of which represents a substantial area of scholarship in its own right. At a minimum, the authors could acknowledge the breadth of the existing literature on these topics and more clearly articulate how the scoping review will contribute to and intersect with these distinct bodies of work.

**Engagement with theoretical perspectives of involvement:**

 As it stands the objectives of the scoping review offers limited potential for advancing theoretical understanding of engaged research, as it does not sufficiently engage with existing theories or conceptual models of PPI. Theory building is constrained when a study does not explicitly build on established theoretical frameworks, yet the authors draw almost exclusively on empirical evidence. As a result, substantial bodies of literature and well-established models of involvement- such as Arnstein’s ladder of participation, Hickey and Kipping’s continuum, Tritter and McCallum’s mosaic model, and Smith et al.’s typology of involvement- are absent from the objectives and focus of the analysis. Similarly, existing models of PPI impact, including those developed by INVOLVE, and GRIP2 reporting guidelines are not considered. This lack of theoretical engagement means that the scoping review proposed in the paper appears conceptually underdeveloped and somewhat isolated from the extensive scholarship on patient involvement across health and social care, research, education, and broader traditions of civic engagement and public participation in public sector decision-making.

**Benefits and beneficiaries of the scoping review:**

 Part of the rationale should be to outline the potential benefits and beneficiaries of this scoping review beyond the immediate benefits to informing the wider study.

**Consistency of aims and objectives:**

 There is inconsistency between the aims described in the abstract and the main body of the paper. The aim as defined in the abstract is to “determine the state of knowledge concerning PPI in mental health research capacity-strengthening initiatives; identify the outcome measures used to evaluate the impact of capacity strengthening initiatives for PPI on contributors, research processes, and policy; and map the barriers and enablers to the implementation of capacity strengthening initiatives for PPI.” The first part of the aim differs from the aim expressed in the body of the paper, which is described as to “describe existing training programmes, onboarding activities, and other skill-building initiatives directed at PPI members in this area, and identify and categorise barriers and enablers to implementing these initiatives.”

 The objectives are then set out to:

 1) Describe the content and implementation processes of capacity-strengthening initiatives utilised to strengthen PPI in mental health research.

 2) Describe outcome measures used to evaluate the experience or impact of the identified capacity-strengthening initiatives on lived experience contributors and broader research or policy implementation goals.

 3) Use the Consolidated Framework for Implementation Research (CFIR) to map identified barriers and enablers of implementing capacity-strengthening initiatives.

 Objective 2 should perhaps be broader to include the outcomes on researchers (skills, professional development, careers), research team dynamics, research environments and cultures.

 Objective 3 would align more closely with the Consolidated Framework for Implementation Research (CFIR) if it explicitly explored all five interrelated CFIR domains. These include intervention characteristics, which encompass features such as perceived complexity, adaptability, and strength of evidence; the outer setting, referring to external influences such as policy contexts, funding requirements, and stakeholder needs; and the inner setting, which captures organisational factors including culture, leadership engagement, resource availability, and readiness for implementation. In addition, the characteristics of individuals domain examines the knowledge, beliefs, skills, and motivations of those involved in implementation, while the process domain focuses on how implementation is planned, enacted, reflected upon, and evaluated over time. Explicit engagement with these domains would strengthen the theoretical coherence of the objective and enhance its contribution to implementation-informed analysis.

**Study Design**

 The authors should justify their selection of CFIR and explain how other implementation frameworks exist and have different purposes e.g., RE-AIM, Theoretical Domains Framework (TDF), PARIHS / i-PARIHS, Normalization Process Theory (NPT), EPIS, Knowledge-to-Action (KTA) Framework.

**Timeframe**

 Both the timeframe for the review and the publication date range of included articles should be explained.

**Best practice frameworks for PPI**

 The authors should consider alignment of the scoping review and outcomes to be considered, with best practice frameworks for PPI, for example, but not limited to:

 Public Involvement Impact Assessment Framework (PiiAF)

https://piiaf.org.uk/

https://pmc.ncbi.nlm.nih.gov/articles/PMC6022311/

 Public Involvement in Research Impact Toolkit (PIRIT)

Public Involvement in Research Impact Toolkit (PIRIT)

 NIHR ARC North East and North Cumbria, Equality, Diversity and Inclusion (EDI) Toolkit

https://arc-nenc.nihr.ac.uk/virtual-college/equality-diversity-and-inclusion-edi-toolkit/

 GRIPP2 reporting framework

https://doi.org/10.1186/s40900-017-0062-2

**Stakeholder consultation**

 The stakeholder consultation phase of the scoping review (as advocated by Levac, Colquhoun, and O’Brien, 2010) could be described in more detail, and a stakeholder engagement framework would provide structure, clarify roles and expectations, and ensure systematic and meaningful involvement of public contributors and other stakeholders throughout the review process. As the authors acknowledge, the field of PPI in mental health research has been shaped by several influential scholars in the UK/Ireland context, whose work provides important context for understanding the significance of the scoping review developed by Anand-Vembar et al. Pioneers such as Diana Rose, Peter Beresford, and Alison Faulkner have emphasized the value of lived experience knowledge, service user leadership, and the ethical and social justice dimensions of involvement. Elizabeth Morrow and Fiona Ross have further advanced critical perspectives on PPI, highlighting issues of power, tokenism, and the need for relational, ethically grounded practices in the context of digital society. Methodologically focused scholars, including Sophie Staniszewska and Kristina Staley, have contributed frameworks and evaluation tools to strengthen the rigor and impact of involvement initiatives. Collectively, these authors provide both the conceptual and practical foundations that inform contemporary efforts to enhance capacity for meaningful PPI in mental health research.

**Methods**

 The scoping review methods are well chosen and referenced. The following points of clarification could enhance the study protocol.

 Definition of mental health: Enhance and reference the definition of mental health research being applied in the scoping review. Expand upon the core concepts to include alternative terms and international variations of terms for PPI, PPIE etc. Expand the involvement concept to include involvement, inclusion, participation, engagement, co-production, co-creation etc. the literature does reveal multiple modes for engagement of users. See: https://onlinelibrary.wiley.com/doi/10.1111/hex.13299?msockid=22180b9dbc9b602f14011e31bd5f61b1

 Clarify which aspects of the research cycle are included – for example, will the scoping review include pre-award involvement e.g., in identifying research priorities, developing tenders or programmes, research commissioning, review, project research, or research evaluation and assessment practices. Or is capacity building limited to only activities within funded research programmes or structures. The scope needs to be clarified for reproducibility.

 Explain inclusion/exclusion of user-led research traditions, such as Survivor research, Advocacy research, Volunteer research, and whether or not these will be included.

 Include hand searching of specific journals: Research Involvement and Engagement, Health Expectations, Research for All, BMJ Open, Patient: Patient-Centered Outcomes Research, BMC Health Services Research, BMC Medicine, International Journal of Integrated Care, Journal of Interprofessional Care, Evidence & Policy.

 Briefly explain ‘Recovery Colleges across Ireland’, for example, grounded in **recovery-oriented and strengths-based principles** **,** Recovery Colleges focus on building knowledge, skills, confidence, and self-management strategies.

**Analysis**

 In the analysis, interrogate where capacity development occurs, such as within research projects or research institutions, or in communities, educational settings, research ready communities, or member-led educational networks such as University of the Third Age (U3A).

 An often overlooked analytic perspective concerns the economic costs and added value of PPI initiatives and their resourcing supports. The review should explicitly consider economic perspectives, including the application of social cost–benefit analysis and social return on investment frameworks, to assess whether and how PPI generates value relative to its resource requirements. Incorporating an analysis of any existing economic evidence, alongside identifying gaps in this evidence, would strengthen the review by highlighting what is currently known about the economics of PPI capacity-building and where further empirical work is needed.

 Provide more detail on who, how and when subject experts will be approached and what they will be asked to contribute to the review and analysis of results. Part of the scoping review methodology is to identify gaps; these experts can usefully provide insight into these gaps.

 Consider ways to analyse diversity, equity and inclusion in PPI capacity development, for example, in terms of accessibility of research leadership opportunities, training programmes and research leadership academies. Specifically considering intersectionality of mental health with personal protected characteristics and PPI. How might marginalised and underserved and underrepresented groups face additional barriers to involvement?

**Dissemination**

 The authors could expand upon the dissemination and communication strategy for the scoping review findings to target audiences. In particular consider producing a policy briefing for research funders and evaluators, recommendations for practice, and plain English summary of the findings for lay audiences.

Is the study design appropriate for the research question?

Yes

Is the rationale for, and objectives of, the study clearly described?

Partly

Are sufficient details of the methods provided to allow replication by others?

Partly

Are the datasets clearly presented in a useable and accessible format?

Not applicable

Reviewer Expertise:

NA

We confirm that we have read this submission and believe that we have an appropriate level of expertise to confirm that it is of an acceptable scientific standard, however we have significant reservations, as outlined above.

# Response

**Published:** 2026-04-07

**Authors:** Shaakya Anand-Vembar

**Affiliations:** Trinity College Dublin School of Nursing and Midwifery, Dublin, Ireland

April 7 ^th^,  2026

 Dear Dr Elizabeth Morrow, Ms. Helen Sloan, Dr Llinos Haf Spencer, and Prof. Mary Lynch,

 We thank you for taking the time to peer review our scoping review protocol and greatly appreciate your expertise and suggestions. Your comments have provided us with important insights that have strengthened the clarity and coherence of the protocol's background, have helped align its objectives and data extraction tool, and have increased its methodological robustness. 

 We have carefully considered the specific points raised in the review, and have addressed them on a point-by-point basis. Please find our responses to your specific comments in this document: https://docs.google.com/document/d/1U8BUFtADN3v5IAUZsJ4xqdU7YVDspYuB/edit?usp=sharing&ouid=107131347417362362861&rtpof=true&sd=true

 Please refer to both the tracked changes in the revised manuscript document, as well as tracked changes in the appendix file. Changes pertaining to your comments have been marked as 'Reviewer 1 Comment __’ in the comments within the revised manuscript, for ease of tracking.

 We hope that the revisions sufficiently address your comments; any suggestions that were found to be out of scope for this review will certainly be considered in the write-up of the final publication. 

 Thank you again for your time and attention,

 Dr Shaakya Anand-Vembar, on behalf of all authors
